# Supplementary material for: Identification of photoperiod-induced specific miRNAs in the adrenal glands of Sunite sheep (Ovis aries)
Source: Front Vet Sci. 2022 Jul 22;9:888207. doi: 10.3389/fvets.2022.888207 (PMC9354845; doi:10.3389/fvets.2022.888207)
Supplement: Supplementary file 1 [file Data_Sheet_1.ZIP › Supplementary file/miRDeep2 file.zip!/miRDeep2 file/Know_Structure/LP42A3/oar-let-7a.pdf]

miRBase precursor : oar-let-7a
Total read count : 305327
oar-let-7a read count : 303776
remaining reads : 1551

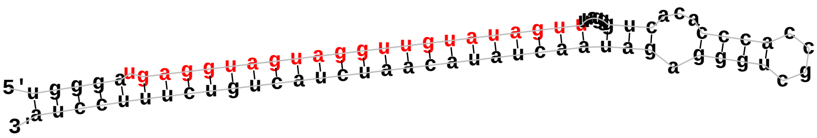

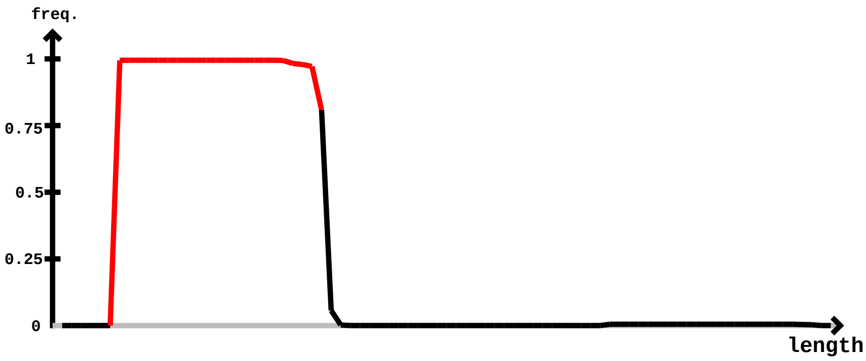

oar-let-7a

```
5'- ugggaugagguaguagguuguauaguuuuagggucacacccaccgcugggagauaacuauacaaucuacugucuuuccua   -3'    exp
    (((((.(((((((((((((((((((((.....(((...((((....)))).)))))))))))))))))))))))))))))    reads      mm         sample
    ....augagguaguagguuguau.........................................................    1          0          seq
    ....augagguaguagguuguauagu......................................................    3          0          seq
    ....Uugagguaguagguuguauaguu.....................................................    9          1          seq
    ....augagguaguagguuguauaguu.....................................................    19         0          seq
    ....augagguaguagguuguauaguuu....................................................    3          0          seq
    ....Uugagguaguagguuguauaguuu....................................................    1          1          seq
    .....ugagguaguagguugua..........................................................    216        0          seq
    .....ugaAguaguagguugua..........................................................    1          1          seq
    .....ugagguagGagguugua..........................................................    24         1          seq
    .....ugagguaguaAguugua..........................................................    5          1          seq
    .....ugagguaguagguuguau.........................................................    2991       0          seq
    .....ugaAguaguagguuguau.........................................................    28         1          seq
    .....ugagguagGagguuguau.........................................................    362        1          seq
    .....ugagguaguaAguuguau.........................................................    22         1          seq
    .....ugagguaguagguuUuau.........................................................    8          1          seq
    .....ugagguUguagguuguau.........................................................    3          1          seq
    .....ugagguagGagguuguaua........................................................    137        1          seq
    .....ugagguaguagguuguaua........................................................    1063       0          seq
    .....ugagguagGagguuguauag.......................................................    358        1          seq
    .....ugagguaguagguuguauag.......................................................    1913       0          seq
    .....ugagguaguagguuguauagu......................................................    42267      0          seq
    .....ugagguagGagguuguauagu......................................................    7425       1          seq
    .....ugagguagGagguuguauaguu.....................................................    23037      1          seq
    .....ugagguaguagguuguauaguu.....................................................    206474     0          seq
    .....ugagguaguagguuguauaguuu....................................................    16629      0          seq
    .....ugagguaguagguuguauaguuuu...................................................    502        0          seq
    .....ugagguaguagguuguauaguuuA...................................................    100        1          seq
    .....ugagguaguagguuguauaguuuua..................................................    1          0          seq
    ......gagguagGagguuguau.........................................................    1          1          seq
    ......gagguaguagguuguau.........................................................    2          0          seq
    ......gagguagGagguuguauagu......................................................    6          1          seq
    ......gagguaguagguuguauagu......................................................    19         0          seq
    ......gagguaguagguuguauaguu.....................................................    115        0          seq
    ......gagguagGagguuguauaguu.....................................................    15         1          seq
    ......gagguaguagguuguauaguuu....................................................    12         0          seq
```

oar-let-7a

```
ugggaugagguaguagguuguauaguuuuagggucacacccaccgcugggagauaacuauacaaucuacugucuuuccua
......gagguaguagguuguauaguuuu...................................................    1      0      seq
........gguaguagguuguauaguu.....................................................    1      0      seq
.........guaguagguuguauagu......................................................    1      0      seq
..........uaguagguuguauaguuuuagg................................................    1      0      seq
...........................uuagggucacacccaccgcu.................................    1      0      seq
...........................uuagggucacacccaccgcugg...............................    1      0      seq
...........................uuagggucacacccaccgcuggg..............................    1      0      seq
...........................uuagggucacacccaccgcuggga.............................    16     0      seq
...........................uuagggucacacccaccgcugggagauaa........................    1      0      seq
....................................................auaacuauacaaucuacugucuuu....    1      0      seq
.....................................................uaacuauacaaucuacugucu......    1      0      seq
.....................................................uaacuauacaaucuacugucuu.....    1      0      seq
.....................................................uaacuauacaaucuacugucuuu....    5      0      seq
......................................................aacuauacaaucuacugucuuuc...    1      0      seq
........................................................cuauacaaucuacuguc.......    1      0      seq
........................................................cuauacaaucuacugucu......    3      0      seq
........................................................cuauacaaucuacugucuu.....    15     0      seq
........................................................cuauacaaucuacugucuuu....    287    0      seq
........................................................cuauacaaucuacuguUuuuc...    1      1      seq
........................................................cuauacaaucuacugucuuuc...    227    0      seq
........................................................cuauacaaucuacugucuuucc..    906    0      seq
........................................................cuauacaaucuacugucuuuccu.    38     0      seq
.........................................................uauacaaucuacugucuuuc...    3      0      seq
.........................................................uauacaaucuacugucuuucc..    6      0      seq
.........................................................uauacaaucuacugucuuuccu.    27     0      seq
..........................................................auacaaucuacugucuuuccu.    7      0      seq
..........................................................auacaaucuacugucuuuccua    1      0      seq
```
